# Is the shock index based classification of hypovolemic shock applicable in multiple injured patients with severe traumatic brain injury?—an analysis of the TraumaRegister DGU^®^

**DOI:** 10.1186/s13049-016-0340-2

**Published:** 2016-12-12

**Authors:** Matthias Fröhlich, Arne Driessen, Andreas Böhmer, Ulrike Nienaber, Alhadi Igressa, Christian Probst, Bertil Bouillon, Marc Maegele, Manuel Mutschler, and the TraumaRegister DGU

**Affiliations:** 1 Department of Orthopaedic Surgery, Traumatology and Sports Traumatology, Cologne-Merheim Medical Centre (CMMC), Witten/Herdecke University, Ostmerheimer Str. 200, D-51109 Cologne, Germany; 2 Institute for Research in Operative Medicine (IFOM), University of Witten/Herdecke, Cologne Merheim Medical Center (CMMC), Ostmerheimerstr.200, D-51109 Cologne, Germany; 3 Department of Anaesthesiology and Intensive Care Medicine, Cologne-Merheim Medical Centre, Witten/Herdecke University, Ostmerheimer Str. 200, D-51109 Cologne, Germany; 4 AUC-Academy for Trauma Surgery, Straße des 17. Juni 106-108, D-10623 Berlin, Germany; 5 Department of Neurosurgery, Cologne-Merheim Medical Centre, Ostmerheimer Str. 200, D-51109 Cologne, Germany; 6 Committee on Emergency Medicine, Intensive Care and Trauma Management of the German Trauma Society (Sektion NIS), Berlin, Germany

**Keywords:** Haemorrhagic shock, Shock index, Traumatic brain injury, Multiple trauma

## Abstract

**Background:**

A new classification of hypovolemic shock based on the shock index (SI) was proposed in 2013. This classification contains four classes of shock and shows good correlation with acidosis, blood product need and mortality. Since their applicability was questioned, the aim of this study was to verify the validity of the new classification in multiple injured patients with traumatic brain injury.

**Methods:**

Between 2002 and 2013, data from 40 888 patients from the TraumaRegister DGU^®^ were analysed. Patients were classified according to their initial SI at hospital admission (*Class I*: *SI* < *0.6, class II: SI ≥0.6 to <1.0, class III SI ≥1.0 to <1.4, class IV: SI ≥1.4).* Patients with an additional severe TBI (AIS ≥ 3) were compared to patients without severe TBI.

**Results:**

16,760 multiple injured patients with TBI (AIS_head_ ≥3) were compared to 24,128 patients without severe TBI. With worsening of SI class, mortality rate increased from 20 to 53% in TBI patients. Worsening SI classes were associated with decreased haemoglobin, platelet counts and Quick’s values. The number of blood units transfused correlated with worsening of SI. Massive transfusion rates increased from 3% in class I to 46% in class IV. The accuracy for predicting transfusion requirements did not differ between TBI and Non TBI patients.

**Discussion:**

The use of the SI based classification enables a quick assessment of patients in hypovolemic shock based on universally available parameters. Although the pathophysiology in TBI and Non TBI patients and early treatment methods such as the use of vasopressors differ, both groups showed an identical probability of recieving blood products within the respective SI class.

**Conclusion:**

Regardless of the presence of TBI, the classification of hypovolemic shock based on the SI enables a fast and reliable assessment of hypovolemic shock in the emergency department. Therefore, the presented study supports the SI as a feasible tool to assess patients at risk for blood product transfusions, even in the presence of severe TBI.

## Background

Severe trauma is the leading cause of death among younger people. Annual deaths worldwide attributed to trauma are expected to increase from five million to more than eight million by 2020 [1]. Despite all improvements in treatment, uncontrolled post-traumatic bleeding is the leading cause of potentially preventable death among these patients [2]. This emphasizes the key role of an early recognition and treatment of haemorrhage, hypovolemia and disorders of coagulation. Therefore, the American College of Surgeons has defined four degrees of hypovolemic shock, which are taught in the Advanced Trauma Life Support (ATLS^®^) training program. These four classes of hypovolemic shock are based upon an estimated blood loss and corresponding vital signs including mental state, blood pressure and pulse rate [3]. Recent analyses from the TraumaRegister DGU^®^ and the TARN registry questioned the classification’s validity [4, 5]. Only 10% of all trauma patients can be classified according to the ATLS^®^ classification [4]. In order to reflect clinical reality more precisely our group proposed a new classification of hypovolemic shock (Table 1) which is based on the shock index (SI) [6]. As the SI is the ratio of heart rate to systolic blood pressure, this index can be immediately calculated when basic vital signs are available. The SI correlates with the extent of hypovolemia and thus may facilitate the early identification of severely injured patients threatened by complications due to blood loss and therefore need urgent treatment, i.e. blood transfusion [6].

**Table 1:** Classification of hypovolemic shock based on the shock index [6]

	Class I	Class II	Class III	Class IV
Shock	no shock	mild shock	moderate shock	severe shock
SI at admission	<0.6	≥0.6 to <1	≥1 to <1.4	≥1.4
Need of blood products	Observe	Consider use of blood products	Prepare transfusion	Prepare massive transfusion

Along with haemorrhage, traumatic brain injury (TBI) deteriorates the outcome and is associated with an increased morbidity and mortality, regardless if it occurs with other injuries or as an isolated mono trauma [7]. In young people, TBI is the leading cause of death and disability [8]. Several studies have shown that TBI in conjunction with haemorrhage might disturb the autonomic response to blood loss or the ability to modulate vascular tone [9–12]. Goldstein described the uncoupling of the autonomic and cardiovascular system [13]. Therefore, the use of vital signs such as heart rate or blood pressure for the assessment of hypovolemic shock has to be questioned. In line with these results, McMahon et al. described the effect of acute TBI on the performance of shock index in a small animal model with combined TBI and haemorrhage [14]. The authors concluded that due to disturbance in the central cardiovascular regulation the SI possibly underestimates the extent of haemorrhage in the presence of acute TBI [14].

Since more than 40% of all severely injured patients in Germany sustain an additional TBI, the aim of this study was to determine if the SI based classification of hypovolemic shock is applicable in the presence of TBI predicting transfusion requirements reliably in patients with and without TBI.

## Methods

### The TraumaRegister DGU^®^

The TraumaRegister DGU^®^ of the German Trauma Society (Deutsche Gesellschaft für Unfallchirurgie, DGU) was founded in 1993. The aim of this multi-centre database is a pseudonymized and standardized documentation of severely injured patients. Detailed presentations of the TraumaRegister DGU^®^ have been published previously [15, 16].

Data are collected prospectively starting at the pre-hospital phase, covering the ED and ICU stay until discharge of the patient. The documentation includes detailed information on demographics, injury pattern, comorbidities, pre- and in-hospital management, course on intensive care unit, relevant laboratory findings including data on transfusion and outcome of each individual. The inclusion criterion is admission to hospital via emergency room with subsequent ICU/ICM care or reaching the hospital with vital signs and death before admission to ICU. Currently, approx. 25,000 cases from more than 600 hospitals are yearly entered into the database.

Scientific data analysis is approved according to a peer review procedure established by the German Trauma Society. The present study is in line with the publication guidelines of the TraumaRegister DGU^®^ and registered as TR-DGU project ID 2011–010.

### Study population

For the present study, datasets entered between 2002 and 2013 into the TraumaRegister DGU^®^ were analysed. In 2002, the online version of the registry was introduced, replacing paper form data collection. Inclusion criteria were age ≥16 years, primary admission, admission to an intensive care unit (ICU) and complete datasets for systolic blood pressure (SBP), heart rate (HR) and Glasgow Coma Scale (GCS). Severe TBI was defined as an AIS_head_ ≥ 3 [17, 18]. The shock index (SI) was calculated for each individual patient by the ratio of HR to SBP at emergency department (ED) admission [6].

In 2013 our group described and validated the shock index as a fast guide to transfusion requirements among a large cohort of multiple trauma patients [6]. With respect to previous descriptions of the SI as a predictor for mortality [19], four classes of SI were defined as follows: *Class I: SI < 0.6–no shock; class II: SI ≥0.6 to <1.0–mild shock; class III: SI ≥1.0 to <1.4–moderate shock and class IV: SI ≥1.4–severe shock* (Table 1) [6].

Further, demographics, injury pattern and vital signs were assessed as present upon ED arrival. Therapeutic interventions such as administration of blood products and intravenous fluids and vasopressors were analysed. Massive transfusion (MT) was defined by the administration of ≥10 blood products (including packed red blood cells (pRBC), fresh frozen plasma (FFP) and thrombocyte concentrates (TC)) within 24 h after ED admission. Coagulopathy was defined by a Quick’s value ≤ 70%, which is equivalent to an international normalized ration (INR) of approximately ≥ 1.3 [20, 21]. Evaluating the reliability of the SI based classification regardless of the injury pattern, patients with an AIS_head_ ≥3 were assigned according to their SI at ED admission and compared to patients without a significant TBI (AIS_head_ ≤2).

### Statistical analysis

Data are presented as means ± 95% confidence interval (CI) for continuous variables or percentages for categorical variables. Formal statistical testing comparing TBI and Non-TBI patients within the respective SI classes was avoided since due to the large sample size even minor differences would result in highly significant results, which could mislead to over-interpretation. The clinical relevance of differences between the observed groups has to be carefully interpreted [22]. For the comparison of the SI based classification in the prediction of transfusion requirements in patients with and without TBI, the area under the receiving operating characteristics curve (AUROC) was calculated with occurrence of transfusion (≥1 blood product) and MT as the state variable. The comparison of two areas under the receiving operating characteristics curve was based upon the 95% confidence interval for each curve. Data were analysed using SPSS statistical software package (Version 21, IBM Inc., Armonk, NY, U.S.A.).

## Results

### Demographics and characteristics

During the observed time period 40 888 patients matched the inclusion criteria. Patients were 46.6 ± 0.2 years old, predominantly male (73%) and severely injured with a mean injury severity score (ISS) of 21.4 ± 0.1. Most patients suffered blunt trauma (95%). Severe head injury, displayed by an AIS ≥ 3, occurred in 41% of the cases (*n* = 16,760). General demographics, injury severities and outcome parameters for the patients are shown in Table 2.

**Table 2:** Patients classified by SI calculated at ED admission and the presence of TBI: demographics, injury mechanism and severities as well as outcome parameters. Continuous variables are presented as mean ± 95% confidence interval; categorical variables are presented as absolute number and percentage

	Class I		Class II		Class III		Class IV	
	SI <0.6		SI ≥0.6 to <1		SI ≥1 to <1.4		SI ≥ 1.4	
	Non-TBI	TBI	Non-TBI	TBI	Non-TBI	TBI	Non-TBI	TBI
Demographics								
n (total, %)	6949 (28.8)	5177 (30.9)	12,780 (53.0)	7861 (46.9)	2015 (8.4)	1741 (10.4)	800 (3.3)	839 (5.0)
Male (n, %)	5324 (77)	3691 (72)	9219 (72)	5678 (72)	1423 (71)	1255 (73)	592 (74)	613 (72)
Age (years; mean ± CI)	48.5 ± 0.5	55 ± 0.6	42 ± 0.3	47 ± 0.5	44 ± 0.8	46 ± 1.0	46 ± 1.3	45 ± 1.4
Blunt trauma (n, %)	6367 (96)	4877 (97)	11,405 (93.1)	7428 (97)	1765 (90)	1605 (95)	702 (91)	768 (94)
Injury Severity								
ISS (points; mean ± CI)	13.3 ± 0.2	24.9 ± 0.3	15.8 ± 0.2	28.4 ± 0.3	23.4 ± 0.6	36.2 ± 0.7	30.6 ± 1.0	43.0 ± 1.1
NISS (points; mean ± CI)	16.4 ± 0.2	33.6 ± 0.4	19.4 ± 0.2	35.7 ± 0.3	28.7 ± 0.6	42.9 ± 0.8	36.7 ± 1.1	49.0 ± 1.1
RISC (points; mean ± CI)	4.3 ± 0.2	23.7 ± 0.7	4.5 ± 0.2	26.4 ± 0.6	11.1 ± 0.8	38.3 ± 1.6	23.9 ± 1.9	52.1 ± 2.4
AIS Thorax ≥3 points (n; %)	2669 (38)	1461 (28)	5554 (44)	3182 (40)	1198 (60)	1021 (59)	541 (68)	585 (70)
AIS Abdomen ≥3 points (n; %)	674 (10)	179 (3)	1985 (15)	661 (8)	606 (30)	357 (21)	355 (44)	286 (34)
AIS Pelvis/Extremities ≥3 points (n; %)	1840 (27)	541 (10)	4554 (36)	1569 (20)	1045 (52)	657 (38)	513 (64)	410 (49)
Outcome								
Mortality (n; %)	154 (2.2)	1057 (20.4)	373 (2.9)	1523 (19.4)	204 (10.1)	637 (36.6)	196 (24.5)	433 (51.6)
Hospital LOS (days; mean ± CI)	16.6 ± 0.4	18.3 ± 0.5	20.4 ± 0.4	21.0 ± 0.5	30.8 ± 1.3	23.0 ± 1.2	31.7 ± 2.2	22.2 ± 1.9
ICU LOS (days; mean ± CI)	4.2 ± 0.2	9.7 ± 0.3	6.1 ± 0.2	11.8 ± 0.3	12.2 ± 0.7	14.7 ± 0.7	15.6 ± 1.2	14.6 ± 1.3
Ventilatior days (days; mean ± CI)	1.8 ± 0.1	6.4 ± 0.3	3.1 ± 0.1	8.1 ± 0.2	7.5 ± 0.6	14.8 ± 0.6	10.8 ± 1.0	11.5 ± 1.1
MOF (n; %)	220 (4)	676 (15)	670 (6)	1393 (20)	302 (17)	483 (32)	186 (28)	273 (41)
Sepsis (n; %)	176 (3)	345 (8)	515 (5)	680 (10)	226 (13)	214 (14)	143 (21)	119 (17)

In all patients, worsening of SI category was associated with an increased ISS, increased in-hospital mortality. Accordingly a higher rate of multiple organ failure (MOF) and sepsis occurred in higher SI classes. Parameters reflecting a complicated treatment such as hospital length of stay (LOS) and ICU (intensive care unit) LOS as well as ventilator-days increased. In all classes, TBI patients showed a higher ISS compared to Non-TBI patients. Within each class, more patients without TBI had injuries associated with high blood loss such as severe abdominal and pelvic injuries. Furthermore, TBI patients had a significantly increased mortality and showed a higher rate of multiple organ failure (MOF) and sepsis.

### Vital signs of TBI patients

As defined a-priori, SBP decreased and HR increased at emergency department admission according to the SI classes. Differences between TBI and Non-TBI patients were not observed as shown in Table 3. The presence of TBI was associated with a lower GCS and remarkable higher pre-clinical intubation rate. However, a higher SI (≥1.4) was associated with lower Glasgow Coma Scale and higher intubation rate in all patients. Table 4 provides the first laboratory findings. Haemoglobin values and platelet counts were lower with worsening SI classes. In the presence of TBI, coagulation markers were more severely impaired compared to Non-TBI patients. In these patients, coagulopathy, that is characterized by a Quick’s value <70% and prolonged aPTT, appeared in class III and IV, assuming a SI of 1.0 or higher.

**Table 3:** Patients classified by SI calculated at ED admission and the presence of TBI: traditional vital signs as presented on scene and at ED admission. Continuous variables are presented as mean ± 95% confidence interval; categorical variables are presented as absolute number and percentage

	Class I		Class II		Class III		Class IV	
	SI <0.6		SI ≥0.6 to <1		SI ≥1 to <1.4		SI ≥ 1.4	
	Non-TBI	TBI	Non-TBI	TBI	Non-TBI	TBI	Non-TBI	TBI
Vital signs								
SBP at scene (mmHg; mean ± CI)	138 ± 0.7	141 ± 1.0	125 ± 0.5	123 ± 0.8	107 ± 1.3	105 ± 1.9	96 ± 2.4	95 ± 2.9
SBP at ED (mmHg; mean ± CI)	149 ± 0.6	149 ± 0.7	126 ± 0.3	123 ± 0.5	97 ± 0.7	96 ± 0.8	71 ± 1.1	70 ± 1.1
HR at scene (beats/min; mean ± CI)	84 ± 0.4	80 ± 0.6	95 ± 0.3	92 ± 0.5	106 ± 1.0	102 ± 1.6	112 ± 2.0	108 ± 2.4
HR at ED (beats/min; mean ± CI)	75 ± 0.3	73 ± 0.4	92 ± 0.3	91 ± 0.4	110 ± 0.8	109 ± 0.9	124 ± 1.4	123 ± 1.4
GCS at scene (points; mean ± CI)	13.9 ± 0.1	9.7 ± 0.1	13.6 ± 0.1	8.8 ± 0.1	12.4 ± 0.2	7.0 ± 0.2	11.2 ± 0.3	6.4 ± 0.3
GCS at ED (points; mean ± CI)	12.5 ± 0.1	8.1 ± 0.1	11.4 ± 0.1	6.7 ± 0.1	8.5 ± 0.2	4.6 ± 0.2	6.2 ± 0.4	3.9 ± 0.2
Intubation rate at ED admission (n; %)	1271 (19)	2497 (49)	3396 (27)	4804 (63)	954 (49)	1398 (81)	528 (68)	716 (87)

**Table 4:** Patients classified by SI calculated at ED admission and the presence of TBI: laboratory findings at ED admission. Continuous variables are presented as mean ± 95% confidence interval

	Class I		Class II		Class III		Class IV	
	SI <0.6		SI ≥0.6 to <1		SI ≥1 to <1.4		SI ≥ 1.4	
	Non-TBI	TBI	Non-TBI	TBI	Non-TBI	TBI	Non-TBI	TBI
Laboratory findings								
Haemoglobin (g/dl; mean ± CI)	13.3 ± 0.1	12.7 ± 0.1	12.8 ± 0.1	12.3 ± 0.1	11.1 ± 0.1	10.8 ± 0.1	9.6 ± 0.2	9.6 ± 0.2
Platelets (tsd/μl; mean ± CI)	222 ± 1	209 ± 2	223 ± 1	208 ± 2	211 ± 4	192 ± 4	190 ± 7	177 ± 5
Quick (%; mean ± CI)	89 ± 0.5	84 ± 0.6	86 ± 0.4	80 ± 0.5	73 ± 1.0	66 ± 1.2	62 ± 1.8	57 ± 2
pTT (seconds; mean ± CI)	28.5 ± 0.2	30.6 ± 0.4	29.2 ± 0.2	32.4 ± 0.4	33.6 ± 0.8	42.7 ± 1.5	44.2 ± 2.1	53.4 ± 2.8
Lactate (mmol/l; mean ± CI)	2.5 ± 0.1	2.5 ± 0.1	2.9 ± 0.1	3.1 ± 0.1	4.3 ± 0.3	4.6 ± 0.4	5.6 ± 0.5	6.4 ± 0.7

### Volume management and transfusion requirements in TBI patients

Volume management and transfusion requirements of TBI patients are displayed in Table 5. With worsening SI, the volume administered and the percentage of patients that received vasopressors increased significantly. According to the predicted transfusion requirements by the TASH score, the observed transfusion incidence increased similarly.

**Table 5:** Patients classified by SI and the presence of TBI: blood products and fluid resuscitation. Continuous variables are presented as mean ± 95% confidence interval; categorical variables are presented as absolute number and percentage

	Class I		Class II		Class III		Class IV	
	SI <0.6		SI ≥0.6 to <1		SI ≥1 to <1.4		SI ≥ 1.4	
	Non-TBI	TBI	Non-TBI	TBI	Non-TBI	TBI	Non-TBI	TBI
Transfusion requirements								
All blood products/units (n; mean ± CI)	0.6 ± 0.1	0.9 ± 0.2	1.8 ± 0.2	2.4 ± 0.2	8.1 ± 0.9	8.3 ± 0.9	17.6 ± 2.0	17.3 ± 1.9
pRBC transfusions/units (n; mean ± CI)	0.5 ± 0.1	0.7 ± 0.1	1.3 ± 0.1	1.6 ± 0.1	4.5 ± 0.4	4.6 ± 0.4	9.1 ± 0.9	8.7 ± 0.8
FFP transfusions/units (n; mean ± CI)	0.3 ± 0.1	0.5 ± 0.1	0.9 ± 0.1	1.3 ± 0.2	3.5 ± 0.4	3.7 ± 0.4	6.9 ± 0.8	6.8 ± 0.8
TC transfusion/units (n; mean ± CI)	0.1 ± 0.0	0.1 ± 0.0	0.1 ± 0.0	0.1 ± 0.0	0.5 ± 0.1	0.5 ± 0.1	1.2 ± 0.2	1.1 ± 0.2
TASH Score (points; mean ± CI)	3.1 ± 0.1	2.9 ± 0.1	4.6 ± 0.1	4.8 ± 0.1	10.1 ± 0.2	10.0 ± 0.2	15.3 ± 0.4	14.6 ± 0.3
IV fliuds at scene (ml; mean ± CI)	814 ± 16	811 ± 18	960 ± 13	1027 ± 18	1237 ± 46	1319 ± 46	1487 ± 81	1522 ± 71
IV fliuds at ED (ml; mean ± CI)	1190 ± 34	1258 ± 40	1582 ± 40	1663 ± 40	2578 ± 119	2479 ± 142	3475 ± 221	3279 ± 213
Vasopressors at scene (n; %)	143 (2)	322 (7)	454 (4)	866 (12)	217 (12)	451 (27)	190 (26)	305 (39)
Vasopressors at ED (n; %)	557 (9)	955 (20)	1746 (14)	2225 (30)	781 (41)	954 (57)	543 (71)	629 (79)

### Comparison of transfusion requirements according to injury characteristics

**Fig. 1:**
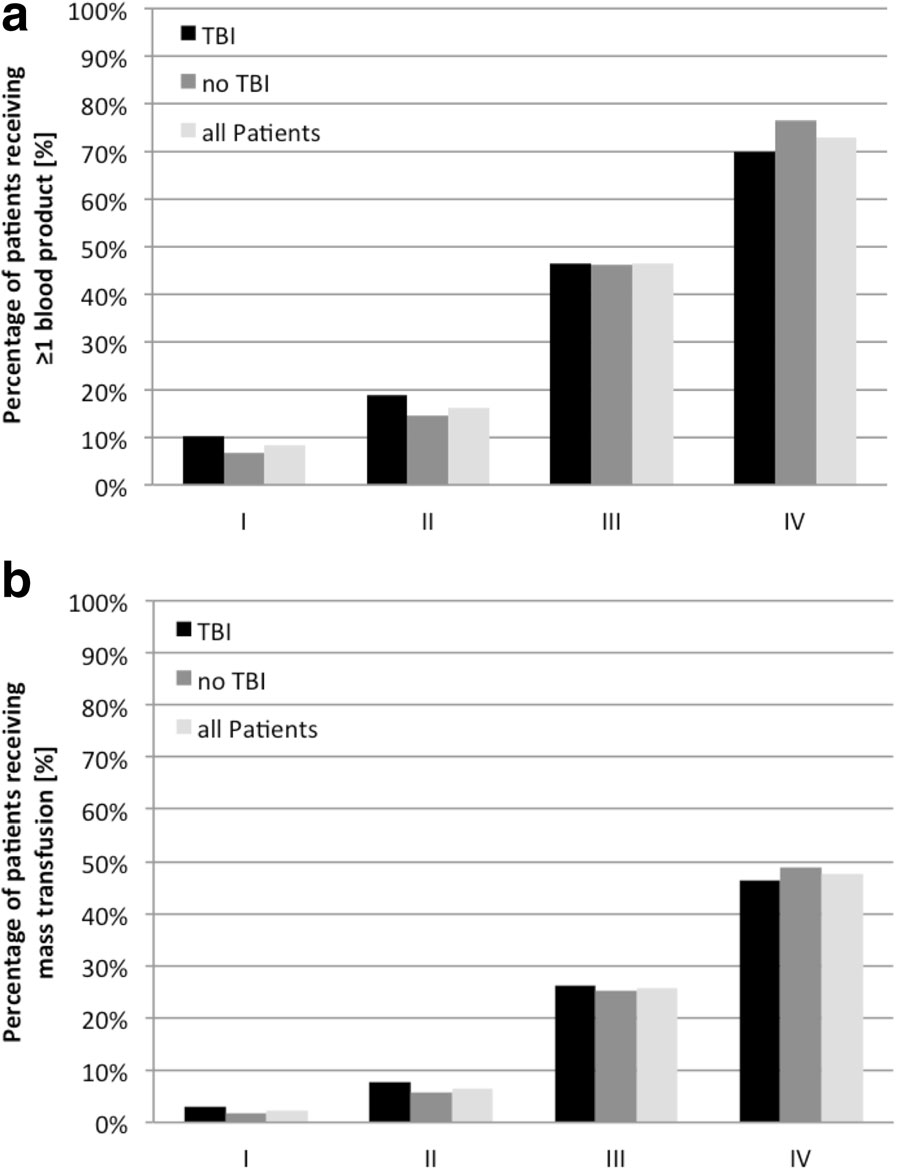
**a** and **b** Ratio of multiple injured patients receiving a) any transfusion or b) mass transfusion regarding the presence of TBI and according to their SI class

The percentage of multiple injured patients receiving at least one blood product or MT increased stepwise from class I to class IV regardless the presence of TBI (Fig. 1). The percentage of TBI patients who received at least one blood product increased from 10% in class I to 70% in class IV. Accordingly the rate of MT increased from 3% in class I to 46% in class IV. In comparison, Non-TBI patients received slightly less blood products in shock classes I and II, whereas the relation turned in class IV with balanced percentages in class III (Fig. 1).

**Fig. 2:**
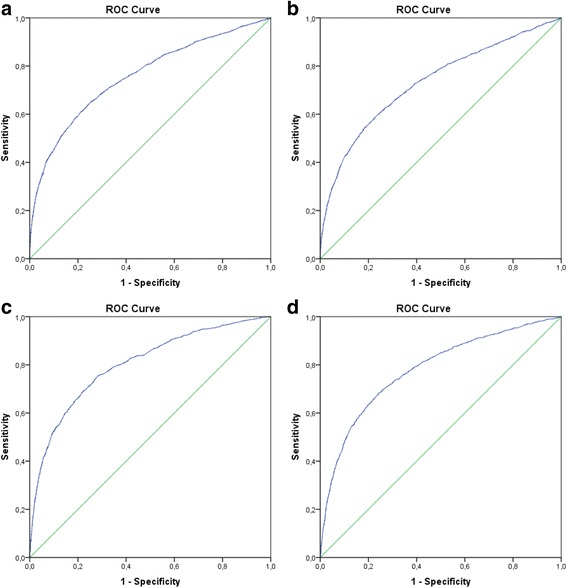
ROC curves displaying the predictive value of the SI as continuous variable regarding the occurrence of transfusion (≥1 blood product; **a** Non-TBI, **b** TBI) and massive transfusion (≥10 blood products; **c**) Non-TBI, **d**) TBI) as state variable

**Fig. 3:**
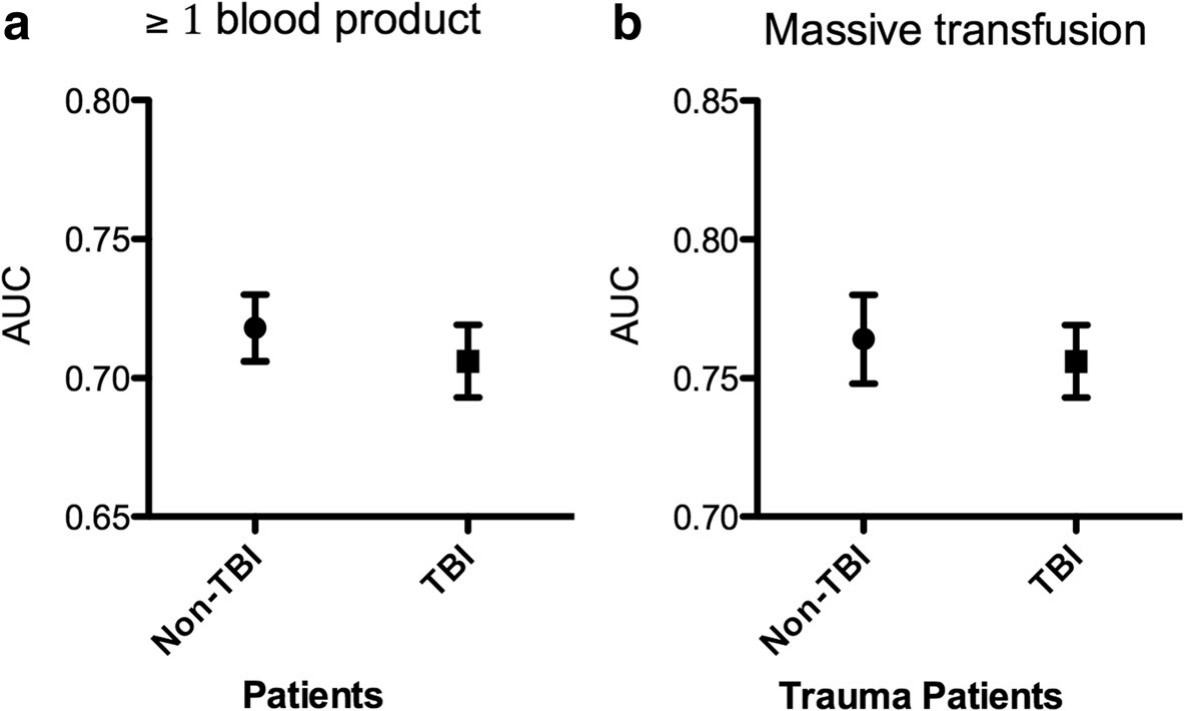
Graphic representation of the performance analysis of the SI regarding **a** any transfusion and **b** massive transfusion displayed as area under the receiver operating curve ± 95% confidence interval

ROC curves displaying the predictive value of the SI regarding the occurrence of transfusion and MT are displayed in Fig. 2. As reflected by an AUROC of 0.706 (0.693–0.719) for TBI patients and 0.718 (0.707–0.730) for Non-TBI patients, the accuracy for predicting the transfusion of ≥1 blood product did not differ significantly (Fig. 3). Accordingly, the accuracy for predicting MT was comparable in both groups (AUROC: TBI 0.756 (0.740–0.773) vs. Non TBI 0.764 (0.748–0.779)).

## Discussion

The SI is a tried and tested approach recognizing the presence of haemodynamic shock. Previously, a shock index based classification has been proposed to assess the extent of hypovolemic shock after trauma in order to realize the need of blood product transfusions [6]. According to the presented nationwide, population-based prospective database analysis, the SI based classification of hypovolemic shock predicts transfusion requirements in trauma resuscitation regardless of the presence of severe TBI.

During the past few years, several approaches targeting the recognition and evaluation of the extent of hypovolemic shock after trauma have been proposed In the present study, we confirmed the SI as a reliable indicator assessing the presence of hypovolemia that is easily ascertained. There is a growing body of evidence, that the SI as the ratio of HR and SBP is more sensitive than its underlying vital signs alone. In a systematic review, Pacagnella assessed the relationship between blood loss and corresponding vital signs [23]. The accuracy in predicting blood loss displayed by the area under receiver operating characteristic curves ranged within the reviewed studies from 0.56 to 0.74 for HR, from 0.56 to 0.79 for SBP and from 0.77 to 0.84 for SI [23]. While an increased prehospital SI has also been shown to be associated with a significant increased risk for MT, the presented results show the SI’s predictive value at ED admission (Fig. 1) [24, 25]. Although the SI is immediately available at admission, the four classes of hypovolemic shock based on SI are equivalent to classifications based on early laboratory findings such as base deficit [6]. Compared to the ATLS^®^ classification, which is a good didactic tool to identify critical patients, the SI based score enables a better prediction for the need of blood products. It proves to be a robust indicator of shock based on readily available clinical variables. However, one key element of ATLS^®^ is its universal and worldwide application, nearly independent of infrastructure and time points of trauma care. The proposed score fulfils these demands, as no blood tests or point of care diagnostics (POCT) are required.

In the present study, regardless of the presence of TBI, an increased SI class was associated with more serious injuries depicted by an increased ISS including higher percentages of thoracic, abdominal and pelvic injuries. This results in a significantly increased mortality according to the respective SI class. Likewise, data from the British Trauma Audit and Research Network (TARN) established the SI among the top markers predicting 48-h mortality [26]. But in difference to our study, Bruijns and colleagues excluded moderate and severe head injuries [26]. Doubtless, severe TBI is associated with increased mortality. But since the incidence of severe TBI was 41% in the presented study population, a clinically useful shock classification should be applicable in all trauma patients including those patients suffering TBI.

Unsurprisingly, TBI patients’ outcome was worse compared to Non-TBI patients’ outcome regardless of the shock class, displayed by ICU LOS, ventilator days and mortality. As we focused on patients with AIS_head_ ≥3, the mortality of 20% in shock classes I and II is in line with previous studies [8], as well as the stepwise increased mortality in Classes III and IV [6, 27]. However, further outcome parameters, which depend on the time of death, such as hospital LOS or ventilator days, decreased from class III to IV in TBI patients, while these parameters increased across all classes from I to IV in the Non-TBI cohort. The combination of severe haemorrhagic shock and TBI, which involves a significantly increased mortality rate and a supposed time of death early after trauma, might explain these differences.

The influence of TBI on the reliability of vital signs and the SI was discussed previously. The uncoupling of the autonomic and cardiovascular system complicates the assessment and usability of blood pressure and heart rate [13]. In an animal model of combined TBI and haemorrhagic shock, the rise of SI was markedly attenuated in non surviving animals which suggests a lack of cardiovascular response to haemorrhage [14]. However, differences were not observed until a blood loss of 40% [14]. McMahon and colleagues concluded that the significantly differing trends in the performance of SI with on-going haemorrhage might lead to an underestimation of the lost blood volume in the presence of acute TBI [14]. However, we could demonstrate that within one shock class the presence of severe TBI did not influence the transfusion frequency. Above a SI ≥ 1.4, blood products and MT were more likely administered to Non-TBI patients, although according to the ROC analysis, the predictive value did not differ between both groups. Therefore, an effect of the observed difference for clinical practice remains questionable. The main goal of the presented classification is to increase awareness and to identify patients at risk for bleeding and to predict reliably the need for blood products.

During the study period, the practice of trauma resuscitation and major transfusion changed considerably [15]. A recent analysis from the TraumaRegister DGU^®^ showed that from 2002–2012 the preclinical administered volume decreased dramatically [15]. At the same time, less severely injured patients received any blood products or MT [15]. Although small volume resuscitation is not recommended in trauma patients with severe TBI, we did not observe any differences in the volume administered. However, the European Guideline on the ‘Management of bleeding and coagulopathy following major trauma” explicitly recommend the same transfusion triggers and Hb-targets for patients with and without TBI [2]. Since this recommendation did not change over the study period [28, 29], differences in the early treatment should not have influenced the results of the presented comparison between TBI and non-TBI patients.

Within the respective SI classes, vasopressors were used more frequently in TBI patients. Regardless of the presence of TBI, vasopressors should be applied cautiously in addition to volume therapy to maintain the target arterial pressure [2]. In patients with a severe TBI a mean arterial pressure ≥ 80 mmHg is recommended while in bleeding Non-TBI patients a systolic blood pressure of 80–90 mmHg should be targeted [2]. This could explain the observed difference between both groups. The increased use of vasopressors influences directly the SI and the consequent SI class by raising the blood pressure. This might result in a false-low SI class and therefore in an underestimation of the extent of hypovolemic shock and the resulting transfusion requirements. However, the demonstrated comparison of applied blood products showed no differences within the respective SI classes (Fig. 1). Therefore, the theoretically false-low SI class due to the use of vasopressors does not seem to influence the applicability of the proposed SI based classification in the rapid assessment of the need of blood products at ED admission.

This study has limitations, as it is a retrospective study of register-data with all the shortcomings associated. We have to rely on recorded data and are not able to verify the validity. The administration of blood products and MT is based on clinicians’ judgments rather than based on an objective measurement of haemorrhage. However, both parameters are well established and serve as surrogates for critical bleeding. We avoided formal statistical testing comparing TBI and Non-TBI patients within the respective SI classes, since due to the large sample size even minor differences would result in highly significant results. This might mislead to over-interpretation and a careful reflection of the differences between the observed groups regarding their clinical relevance is needed. In spite of these restrictions, we are confident that the SI based classification is a feasible tool to assess patients in hypovolemic shock and at risk for blood product transfusions.

## Conclusion

Summarizing, the proposed classification of hypovolemic shock based on the SI proved to perform equally in multiple injured trauma patients with and without severe TBI. Within the four classes of hypovolemic shock, no clinical relevant differences in transfusion requirements between TBI and non-TBI patients were observed. Therefore, the presented study confirmed that the SI based classification is an easy and reliable tool to identify trauma patients at risk for the need of blood products regardless of the presence of TBI.

## Abbreviations

95% CI: 95% confidence interval
AIS: Abbreviated injury scale
ATLS^®^: Advanced trauma life support
AUROC: Area under the receiving operating characteristics curve
ED: Emergency Department
GCS: Glasgow coma scale
HR: Heart rate
ICU: Intensive care unit
INR: International normalized ratio
ISS: Injury severity score
LOS: Length of stay
MOF: Multiple organ failure
MT: Massive transfusion
NISS: New injury severity score
POCT: Point of care diagnostics
PTT: Partial thromboplastin time
RISC: Revised injury severity classification
SBP: Systolic blood pressure
SI: Shock Index
TASH score: Trauma associated severe haemorrhage score
TBI: Traumatic brain injury
